# Is the Aligning Prism Measured with the Mallett Unit Correlated with Fusional Vergence Reserves?

**DOI:** 10.1371/journal.pone.0042832

**Published:** 2012-08-08

**Authors:** Miriam L. Conway, Jennifer Thomas, Ahalya Subramanian

**Affiliations:** Division of Optometry and Visual Science, City University, London, United Kingdom; Univeristy of Melbourne, Australia

## Abstract

**Background:**

The Mallett Unit is a clinical test designed to detect the fixation disparity that is most likely to occur in the presence of a decompensated heterophoria. It measures the associated phoria, which is the “aligning prism” needed to nullify the subjective disparity. The technique has gained widespread acceptance within professions such as optometry, for investigating suspected cases of decompensating heterophoria; it is, however, rarely used by orthoptists and ophthalmologists. The aim of this study was to investigate whether fusional vergence reserves, measured routinely by both orthoptists and ophthalmologists to detect heterophoria decompensation, were correlated with aligning prism (associated phoria) in a normal clinical population.

**Methodology/Principal Findings:**

Aligning prism (using the Mallett Unit) and fusional vergence reserves (using a prism bar) were measured in 500 participants (mean 41.63 years; standard deviation 11.86 years) at 40 cm and 6 m. At 40 cm a strong correlation (p<0.001) between base in aligning prism (Exo FD) and positive fusional reserves was found. Of the participants with zero aligning prism 30% had reduced fusional reserves. At 6 m a weak correlation between base out aligning prism (Eso FD) and negative fusional reserves was found to break (p = 0.01) and to recovery (p = 0.048). Of the participants with zero aligning prism 12% reported reduced fusional reserves.

**Conclusions/Significance:**

For near vision testing, the strong inverse correlation between base in aligning prism (Exo FD) and fusional vergence reserves supports the notion that both measures are indicators of decompensation of heterophoria. For distance vision testing and for those patients reporting zero aligning prism further research is required to determine why the relationship appears to be weak/non-existent?

## Introduction

When an object is viewed during normal binocular viewing conditions, then the image falls upon the same fixation point (fovea) of both eyes. Sensory fusion allows the two images, falling on corresponding retinal points, to be interpreted as one resulting in normal binocular single vision. Heterophoria is defined as the tendency for the two visual axes of the eyes not to be directed towards the point of fixation, in the absence of an adequate stimulus to fusion [1]. Heterophoria decompensation is defined as any heterophoria which gives rise to symptoms or to suppression [1]. For a heterophoria to remain compensated Sheard’s criterion states that the fusional reserve opposing the heterophoria, should be at least twice the size of the heterophoria [2]. A patient with a 10 dioptre esophoria should therefore have at least 20 dioptres of base in fusional reserve to be able to overcome the heterophoria. Percival’s Criterion states that for the heterophoria to remain compensated the larger horizontal fusional reserve should be no more than twice the size of the smaller [3]. A patient with 20 dioptres base out of fusional reserve should therefore have no less than 10 dioptres base in reserve to be able to overcome the heterophoria. If the fusional reserve is insufficient then the heterophoria will decompensate into a heterotropia giving rise to symptoms and/or a reduction in the quality of that patient’s binocular vision. Other factors which may lead to decompensation include the size of the deviation and or reduction in sensory fusion usually as a result of uncorrected refractive error or pathology.

Fixation disparity is said to present if there is a misalignment of the visual axes (disparity) which is sufficiently small for the retinal image to fall upon corresponding retinal locations preserving binocular single vision [4]. The Mallett Unit is a clinical test that it is designed to detect the fixation disparity that is most likely to occur when there is a decompensated phoria [5]. The Mallett Unit does not quantify fixation disparity itself, instead it measures the associated phoria which is the “aligning prism” that nullifies the subjective disparity [5]. Research has shown that patients with associated phoria report either decreased binocular cortical responses [6], symptoms attributable to decompensating heterophoria at near [7]–[8], reduced binocular visual acuity [9], reduced reading speed [10] and elevated contrast sensitivity thresholds [11]. Under conditions of tiredness [12] and low illumination [13] an increase in both the degree of associated phoria and the levels of reported symptoms has been documented.

The Mallett Unit has gained widespread acceptance within certain professions such as optometry, for investigating suspected cases of decompensating heterophoria as it is quick to administer, inexpensive, non invasive and avoids the need for measuring eye position objectively. Text books written by optometrists suggest that symptomatic heterophoria should be investigated by assessing the size and stability of the Mallett aligning prism and then measuring fusional reserves to assess further if the results from the Mallett Unit are borderline [5]. In other professions such as orthoptics and ophthalmology the technique is rarely used. Text books written by these professions suggest that fusional vergence reserves should be routinely assessed when investigating heterophorias, however the Mallett Unit is not named as a potential clinical test [14]–[16]. In fact von Noorden (2002) [16] goes further to state that the available evidence is insufficient to establish that fixation disparity is anything more than a physiological variant of normal binocular vision. If aligning prism is a measure of decompensation then in a person with good sensory fusion (corrected refractive error, no significant anisometropia and no pathology) it is reasonable to hypothesise that the Mallett Unit should show a reasonable correlation with fusional reserves.

The aim of this study was to investigate if commonly used clinical measures of fusional reserves and aligning prism are well related. If they are it provides further support to clinicians who have previously failed to include the Mallett Unit as a method of investigating decompensating phoria. It also supports the idea that either measure may be used in practice depending on which is most appropriate for the patient and available within the clinic.

## Materials and Methods

Five hundred participants were recruited from an optometry practice. Exclusion criteria included ocular pathology, visual acuity less than 6/6, anisometropia greater than 2 dioptres, history of orthoptic exercises, strabismus or a prism in their current prescription. Patients were initially refracted using standard objective and subjective techniques. If any prescription was required that was going to be prescribed to the patient for everyday use then it was worn in a trial frame for the rest of the examination for distance or near as required.

**Figure 1:**
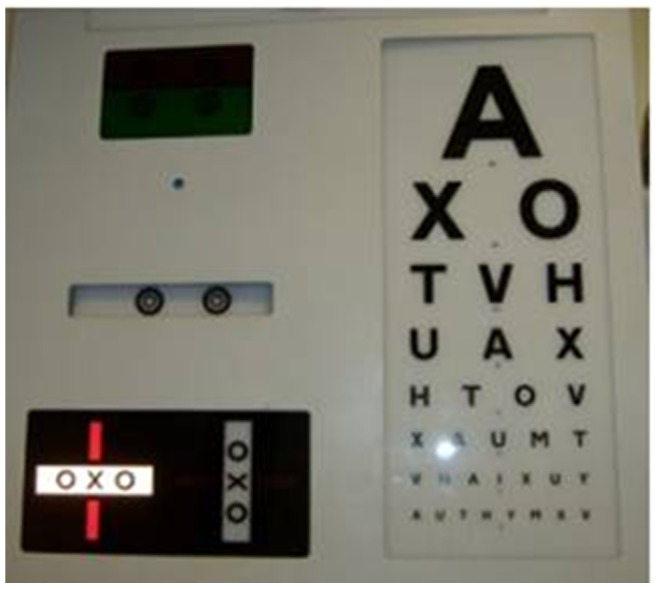
A picture of the distance Mallett Unit (bottom left rectangle).

The order the tests were carried out in was fixed, as it has been shown that carrying out dissociating procedures before measuring aligning prism can increase the aligning prism measured, in patients with unstable binocular vision [17]. During history and symptoms the patient was asked about any symptoms they were experiencing. If they reported symptoms of blurred vision, aching eyes, double vision, distortions, monocular comfort (wanting to close or cover one eye), tired eyes, general irritation or headaches related to completely visual tasks they were recorded as symptomatic. The distance at which these symptoms were reported was recorded. A cover test was performed to rule out any heterotropia which excluded the patient from the study. Aligning prism was first measured at a distance of 6 m using a distance Mallett Unit on a back lit wall mounted chart with a mirror (see Figure 1). The horizontal aligning prism was then measured at 40 cm using the near Mallett Unit (Mallett 1964/1999). Both units contain a central fusion lock which is known to be important when measuring aligning prism [18]–[20]. All patients received instructions to carry out the test based on recommendations by Karania & Evans (2006) [8] as it has been shown that appropriate instructions result in more likelihood of picking up symptomatic patients. A prism bar cover test was performed using a Gulden prism bar (Gulden Ophthalmics) at a distance of 6 m and 40 cm to measure the heterophoria at distance and near respectively. Fusional reserves were measured using a Gulden prism bar. The fixation target was a line of letters equivalent to a Snellen visual acuity of 6/9. The prism was increased by one increment of the prism bar every two seconds. The patient was instructed to concentrate on looking at the target and to report any blurring or doubling of the target. The patient was instructed not to force their eyes but to look normally at the target throughout the test. Blur, break and recovery points were recorded. If the patient could not detect a blur point this was recorded by an X. The blur point measures the limits which accommodation can clear the fixation image due to an increase in vergence demand. When the divergence reserves are measured at near accommodation relaxes to provide additional divergence in order to prevent diplopia, resulting in blur. When the divergence range is measured in the distance blur is not recorded because accommodation is already relaxed and the break point is obtained without blur [21]. Sheard’s and Percival’s values were then calculated for each patient. Sheard’s Value was calculated as the fusional reserve opposing the heterophoria to blur point (or, if no blur to break point) divided by the heterophoria. For example a patient with an exophoria measuring 10 prism dioptres and fusional reserves measuring 20 prism dioptres base out would actually have a Sheard’s Value equal to 2. Percival’s criterion was only used at near as normal distance fusional reserves would not satisfy Percival’s criterion. Percival’s Value was calculated as the base out fusional reserve to break divided by the base in fusional reserve to break. For example if the base out fusional reserve to break measured 20 prism dioptres and the base in fusional reserve to break measured 10 prism dioptres then Percival’s Value equals 2. The same optometrist carried out all investigations. The study was approved by City University London’s Research and Ethical Committee and the study adhered to the tenets of the declaration of Helsinki. Written informed consent was obtained from the subjects after explanation of the nature and possible consequences of the study.

## Results

All five hundred participants completed the study and their age ranged between 18 and 59 years (mean 41.63 years, standard deviation (SD) 11.86 years). All data was recorded with esophoric deviations labelled as positive values and exophoric deviations labelled as negative values. Heterophoria at 40 cm ranged from 20Δ of exophoria to 6Δ of esophoria with a mean of 5.85Δ of exophoria (SD 3.20). At 6 m heterophorias ranged from 10 prism dioptres (Δ) of exophoria to 6Δ of esophoria with a mean of 1.56Δ of exophoria (SD 3.25). The mean and SD for the fusional vergence reserves are recorded in Table 1. At 40 cm, 299 (60%) had no aligning prism and at 6 m, 373 participants (75%) had no aligning prism. Out of the 299 participants who had no aligning prism at near; 107 (36%) complained of visual symptoms at near, 89 (30%) failed to meet Sheard’s Criterion and (157) 53% failed to meet Percival’s Criterion at near. Out of the 373 participants who had no aligning prism in the distance; 77 (21%) complained of visual symptoms in the distance and 43 (12%) failed to meet to Sheard’s Criterion in the distance.

**Table 1:** The mean and SD of the participants’ fusional reserves.

Fusional reserve	Mean (Δ)	SD
Negative to break at 6 m	8.78	2.33
Negative to recovery at 6 m	6.19	1.86
Positive to blur at 6 m	9.84	4.21
Positive to break at 6 m	20.75	6.44
Positive to recovery at 6 m	11.71	3.84
Negative to blur at 40 cm	9.99	3.30
Negative to break at 40 cm	13.29	4.49
Negative to recovery at 40 cm	10.50	4.04
Positive to blur at 40 cm	18.31	6.74
Positive to break at 40 cm	27.06	8.24
Positive to recovery at 40 cm	19.32	7.10

**Figure 2:**
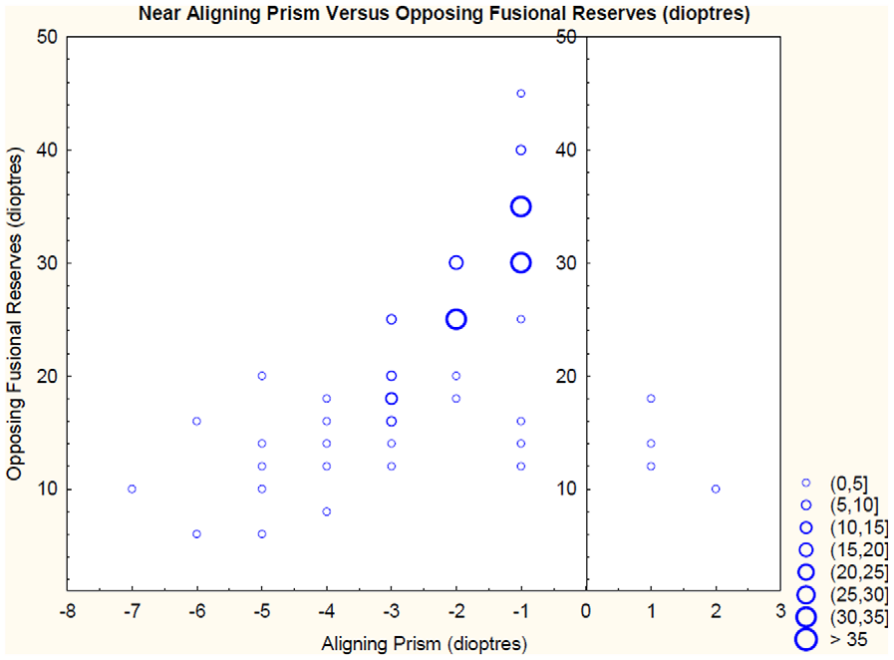
A scattergraph of opposing fusional reserves to break versus aligning prism at 40 cm.

**Figure 3:**
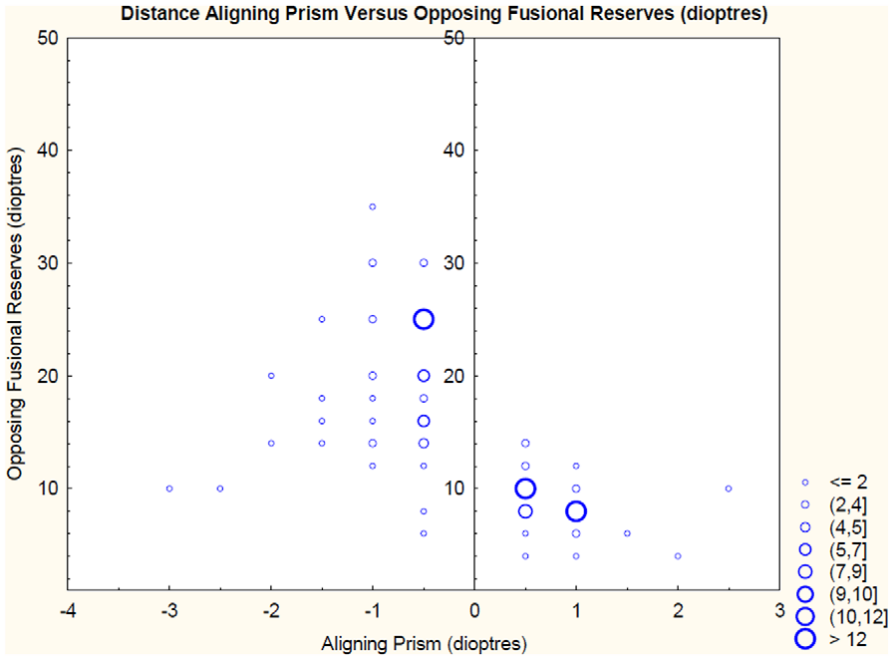
A scattergraph of opposing fusional reserves to break versus aligning prism at 6 m.

A correlation of the fusional reserve that acts against the direction of the aligning prism was carried out for blur, break and recovery. Additionally, a correlation of both Sheard’s Value and Percival’s Value versus aligning prism was carried out. Correlations were carried out for both near and distance and for the group of patients with symptoms attributed to visual tasks and the entire group independent of symptomatic status (Table 2 and 3). A scattergraph of aligning prism versus opposing fusional amplitude (to break) for both near and distance for the entire group of patients is presented in Figure 2 and 3.

**Table 2:** Correlation results for the entire group (Z = insufficient numbers for the calculation).

Entire group	R	R square	Adjusted R square	P value
Near Exo FD Vs BO reserves to blur (n = 197)	0.708	0.501	0.498	<0.001
Near Exo FD Vs BO reserves to break (n = 197)	0.812	0.660	0.658	<0.001
Near Exo FD Vs BO reserves to recovery (n = 197)	0.766	0.587	0.585	<0.001
Near Exo FD Vs Sheard’s Value (n = 197)	0.429	0.184	0.180	<0.001
Near Exo FD Vs Percival’s Value to break (n = 197)	0.301	0.096	0.092	<0.001
Near Eso FD Vs BI reserves to blur (n = 4)	Z	Z	Z	Z
Near Eso FD Vs BI reserves to break (n = 4)	Z	Z	Z	Z
Near Eso FD Vs BI reserves to recovery (n = 4)	Z	Z	Z	Z
Near Eso FD Vs Sheard’s Value (n = 4)	Z	Z	Z	Z
Near Eso FD Vs Percival’s Value to break (n = 4)	Z	Z	Z	Z
Distance Exo FD Vs BO reserves to blur (n = 70)	NS	NS	NS	NS
Distance Exo FD Vs BO reserves to break (n = 70)	NS	NS	NS	NS
Distance Exo FD Vs BO reserves to recovery (n = 70)	NS	NS	NS	NS
Distance Exo FD Vs Sheard’s Value (n = 70)	0.295	0.087	0.073	p = 0.013
Distance Eso FD Vs BI reserves to break (n = 57)	0.340	0.116	0.099	p = 0.01
Distance Eso FD Vs BI reserves to recovery (n = 57)	0.263	0.069	0.052	p = 0.048
Distance Eso FD Vs Sheard’s Value (n = 57)	NS	NS	NS	NS

**Table 3:** Correlation results for the symptomatic group (Z = insufficient numbers for the calculation).

Symptomatic group	R	R square	Adjusted R square	P value
Near Exo FD Vs BO reserves to blur (n = 141)	0.722	0.521	0.518	<0.001
Near Exo FD Vs BO reserves to break (n = 141)	0.820	0.673	0.671	<0.001
Near Exo FD Vs BO reserves to recovery (n = 141)	0.793	0.629	0.627	<0.001
Near Exo FD Vs Sheard’s Value (n = 141)	0.568	0.322	0.318	<0.001
Near Exo FD Vs Percival’s Value to break (n = 141)	0.358	0.128	0.122	<0.001
Near Eso FD Vs BI reserves to blur (n = 2)	Z	Z	Z	Z
Near Eso FD Vs BI reserves to break (n = 2)	Z	Z	Z	Z
Near Eso FD Vs BI reserves to recovery(n = 2)	Z	Z	Z	Z
Near Eso FD Vs Sheard’s Value (n = 2)	Z	Z	Z	Z
Near Eso FD Vs Percival’s Value to break (n = 4)	Z	Z	Z	Z
Distance Exo FD Vs BO reserves to blur (n = 17)	NS	NS	NS	NS
Distance Exo FD Vs BO reserves to break (n = 17)	NS	NS	NS	NS
Distance Exo FD Vs BO reserves to recovery (n = 17)	NS	NS	NS	NS
Distance Exo FD Vs Sheard’s Value (n = 17)	NS	NS	NS	NS
Distance Eso FD Vs BI reserves to break (n = 11)	NS	NS	NS	NS
Distance Eso FD Vs BI reserves to recovery (n = 11)	NS	NS	NS	NS
Distance Eso FD Vs Sheard’s Value (n = 11)	NS	NS	NS	NS

At 40 cm the correlation between base in aligning prism (Exo FD) and all variables was found to be statistically significant p<0.001 (Table 2). The adjusted R square values for base in aligning prism (Exo FD) against positive fusional amplitudes were 49.8% to blur, 65.8% to break, 58.5% to recovery, 18% for Sheard’s Value and 9.2% for Percival’s Value. These correlations indicate that a patient with a large base in aligning prism is likely to have poor positive fusional reserves. Additionally, when all asymptomatic patients were removed from the calculation (Table 3) the adjusted R square values for base in aligning prism (Exo FD) against positive fusional amplitudes increased to 51.8% to blur, 67.1% to break, 62.7% to recovery, 31.8% for Sheard’s Value and 12.2% for Percival’s Value There were insufficient numbers to accurately carry out a correlation between base out aligning prism (Eso FD) and negative fusional reserves at 40 cm.

At 6 m the correlations of base out aligning prism (Eso FD) measured against negative fusional amplitudes to break R = 0.340 (p = 0.01) and to recovery R = 0.263 (p = 0.048) were statistically significant (Table 2). Adjusted R square values were 0.099 and 0.052 to break and recovery respectively, so less than 10% of the variation in the fusional reserves were related to the change in aligning prism. Additionally, when all asymptomatic patients were removed from the calculation (Table 3) the correlation of base out aligning prism at 6 m measured against negative fusional reserves failed to reach statistical significance. This was probably at least partly explained by the reduction in numbers from 57 participants down to 11. In the distance a weak but statistically significant correlation between base in aligning prism (Exo FD) and Sheard’s Value was also documented (R = 0.295 (p = 0.013); adjusted R square  = 0.073) however, only for the entire group. No other statistically significant relationships were found in the distance. Additionally, when the results were Bonferroni adjusted to allow for multiple comparisons none of the correlations in the distance reached statistical significance.

## Discussion

When a base in aligning prism (Exo FD) was present at near the size of the aligning prism was a good correlate of opposing fusional amplitude to blur, break and recovery, in both the symptomatic group of patients and the entire group (p<0.001). This finding is unsurprising in light of earlier research which documents that an aligning prism of ≥1 dioptres in pre-presbyopes and ≥2 dioptres in presbyopes at near is likely to be associated with symptoms attributed to decompensating heterophorias [7]. In fact correcting an aligning prism of ≥2 dioptres has been shown to significantly improve near binocular visual acuity [9], distance binocular visual acuity [22] or reading speed measured via Wilkins rate of reading test [10]. At near the only correlation which produced a small (weak) effect size with base in aligning prism (Exo FD) in both the symptomatic group (12.2%) and the entire group (9.2%) was Percival’s Value. This finding is also predictable as previous research has shown that it was Sheard’s Criterion and not Percival’s Criterion that was a good discriminator of symptomatic exo deviations [23]–[24].

At distance (6 m) only three correlations reached statistical significance when all the subjects were included regardless of symptoms: base in aligning prism (Exo FD) vs Sheard’s Criteria and base out aligning prism (Eso FD) vs negative fusional reserves to break and recovery. In all three conditions less that 10% of the variation in fusional reserves was related to aligning prism. Furthermore when results were Bonferoni adjusted to allow for multiple comparisons none of the correlations in the distance reached statistical significance. In a clinical setting it would therefore be unreliable to assume that fusional reserves and aligning prism were closely related at 6 m. Our findings might be explained in part by the type of distance Mallett Unit used as researchers have shown that inclusion of a peripheral fusion lock might improve detection of fixation disparity [18]. Alternatively, it might be because the current study failed to use a validated symptom questionnaire such as the (CISS) [25]. With the chosen methodology it is possible that some of the symptoms may have been attributed to other ocular factors and a more thorough assessment may have revealed a stronger correlation between the symptomatic group and fusional reserves. Future studies should therefore include a system which grades both the quantity and severity of symptoms using a validated symptom questionnaire. It is of interest to note however that despite these limitations the present data again supports the findings from earlier research which suggests that an aligning prism reported in the distance is not a good predictor of binocular vision symptoms suggestive of a decompensating heterophoria [7]–[8], [26]–[27]. In fact the only research to support the use of the Mallett Unit in the distance, reported that correction of the associated phoria resulted in an improvement the equivalent of one half of a line on the Snellen chart (approximately 0.05 logMAR) [22]. However, the authors acknowledged that in clinical terms this difference was small. Evans (2010) [28] suggests one possible reason for the lack of correlation between distance aligning prism and symptoms is attributable to the different nature of decompensation at near and in the distance. This hypothesis requires further investigation.

Overall results suggest that aligning prism, measured via the Mallett Unit is inconsistently related to fusional vergence reserves. The technique shows a good agreement with fusional vergence reserves at near provided a base in aligning prism (Exo FD) is documented. Further research is needed to determine if those patients with reduced fusional vergence reserves and zero aligning prism at near are at risk from heterophoria decompensation in the future. Thirty six percent (107/299) of participants with zero aligning prism complained of visual symptoms at near whilst thirty percent failed to meet Sheard’s Criterion and fifty three percent failed to meet Percival’s Criterion. Research is also required to validate the relationship between a base out aligning prism (Eso FD) at near and opposing fusional reserve, however, it would require several thousand participants in a normal population to achieve satisfactory numbers of participants with a base out aligning prism (Eso FD). In the distance the technique shows at best a weak correlation between base out aligning prism and negative fusional reserves, suggesting that the two techniques are not closely related in the distance.

Present findings partially explain why this clinical test remains contentious despite several decades of research. For near vision testing, the strong inverse correlation between a base in aligning prism (Exo FD) and fusional vergence reserves suggests both measures are indicators of heterophoria decompensation. For distance vision testing, and when zero aligning prism is reported further research is required to understand why the relationship is weak/non-existent?
